# Whole-Genome Sequence of Multidrug-Resistant Klebsiella pneumoniae MNH_G2C5, Isolated from Bovine Clinical Mastitis Milk

**DOI:** 10.1128/mra.00079-23

**Published:** 2023-04-24

**Authors:** M. Nazmul Hoque, Zannatara Moyna, Golam Mahbub Faisal, Ziban Chandra Das, Tofazzal Islam

**Affiliations:** a Molecular Biology and Bioinformatics Laboratory, Department of Gynaecology, Obstetrics and Reproductive Health, Bangabandhu Sheikh Mujibur Rahman Agricultural University (BSMRAU), Gazipur, Bangladesh; b Institute of Biotechnology and Genetic Engineering, Bangabandhu Sheikh Mujibur Rahman Agricultural University, Gazipur, Bangladesh; Indiana University, Bloomington

## Abstract

Klebsiella pneumoniae is one of the most common and important mastitis-causing bacteria, and strain MNH_G2C5 was isolated from the milk of a cow suffering from clinical mastitis in a dairy farm of the Gazipur district of Bangladesh. The MNH_G2C5 genome was estimated to be 4,589,728 bp, with 65.5% genome coverage.

## ANNOUNCEMENT

Klebsiella pneumoniae is one of the major pathogens causing clinical mastitis (CM) (1), a severe and prolonged intramammary infection (2, 3) that is often accompanied by lower milk production (4, 5). This opportunistic pathogen uses very efficient strategies to override host immunity in order to colonize and invade mammary tissues and then causes episodes of CM (6). Widespread use and misuse of antibiotics have led to the emergence of antimicrobial resistance in bacteria with different multidrug-resistant clones, which is a worldwide public health concern (7, 8). In past decades, whole-genome sequencing (WGS) has developed rapidly and revolutionized the field of infectious diseases in both humans and animals (9, 10).

For isolation and phenotypic identification of this bacterium, CM milk samples were streaked on MacConkey agar (Oxoid; Thermo Fisher Scientific, UK) plates and incubated at 37°C for 24 h (3, 11). The pathogen was identified based on colony morphology, Gram staining, and biochemical tests such as indole, methyl red, and citrate (IMViC) tests and lactose fermentation on MacConkey agar (3). Further identification and confirmation of the isolates were carried out by 16S rRNA gene sequencing (3). The confirmed K. pneumoniae strain MNH_G2C5 was further grown in nutrient broth (Biolife Italiana, Italy) at 37°C for 24 h, and finally genomic DNA was extracted from the broth culture using the QIAamp DNA minikit (Qiagen, Hilden, Germany). The concentration and quality of DNA were determined using a NanoDrop 2000 UV-visible spectrophotometer (Thermo Fisher Scientific, Waltham, MA, USA). DNA libraries were prepared using the Nextera DNA Flex library preparation kit (Illumina, San Diego, CA, USA) according to the manufacturer’s protocol, and WGS of the prepared libraries was performed with an Illumina MiSeq sequencer (Illumina) using a paired-end (2 × 250 bp) protocol. The paired-end raw reads (*n* = 7,492,958 bp) were trimmed using Trimmomatic v0.39 (12) (with parameters leading:20, slidingwindow:4:20:20, trailing:20, minlen=36) to remove Illumina adapters, known Illumina artifacts, and phiX and were quality checked using FastQC v0.11.7 (https://www.bioinformatics.babraham.ac.uk/projects/fastqc). Reads with Phred scores of >20 were assembled *de novo* using SPAdes v3.9.0 (13), and the quality of the assembled genome was checked using QUAST v5.0.2 (14). Genome annotation was performed using the NCBI Prokaryotic Genome Annotation Pipeline (PGAP) v6.4 (15). Antimicrobial resistance repertoires were determined using the ResFinder v4.0 database (16), while the PlasmidFinder (17), Virulence Factor Database (VFDB) (18), and RAST FIGfams v70 (19) databases were used to predict plasmids, virulence factors, and metabolic functions, respectively, in the draft genome. Default parameters were used for all software unless otherwise specified.

The assembled genome of MNH_G2C5 had 65.5% genome coverage and possessed 36 contigs. The annotated chromosome length, GC content, and *N*_50_ value of the assembled genome were 4,589,728 bp, 57.7%, and 149,790 bp, respectively. The lengths of the largest and smallest contigs were 922,712 and 13,736 bp, respectively. The MNH_G2C5 genome contained 341 subsystems, 4,475 protein-coding sequences, and 40 RNA genes. Thirty-seven antimicrobial resistance genes, conferring resistance to multiple antibiotics and metals, and 92 virulence factors were predicted in the draft genome. One plasmid replicon (i.e., IncR_pKPN) of 48,149 bp was identified in the genome (with 95% identity and 60% coverage).

The Animal Research Ethics Committee (AREC) of the Bangabandhu Sheikh Mujibur Rahman Agricultural University reviewed and approved the study under reference number FVMAS/AREC/2023/6679 (16 January 2023).

### Data availability.

The WGS shotgun project for K. pneumoniae MNH_G2C5 was deposited in NCBI/GenBank under the accession number JAQLOG000000000, and the raw reads were deposited under SRA accession number SRR23757280 (BioProject accession number PRJNA923580). The version described in this paper is version JAQLOG010000000.
